# β-Adrenoceptors Trigger Melatonin Synthesis in Phagocytes

**DOI:** 10.3390/ijms19082182

**Published:** 2018-07-26

**Authors:** Marco A. Pires-Lapa, Claudia E. Carvalho-Sousa, Erika Cecon, Pedro A. Fernandes, Regina P. Markus

**Affiliations:** 1 Laboratory of Chronopharmacology, Deartment of Physiology, Institute of Bioscience, University of São Paulo, 05508-900 São Paulo, Brazil; marco_lapa_bio@yahoo.com.br (M.A.P.-L.); claudiabaete@gmail.com (C.E.C.-S.); kika.cecon@gmail.com (E.C.); 2 Laboratory of Neuroimmunoendocrinology, Department of Physiology, Institute of Bioscience, University of São Paulo, 05508-900 São Paulo, Brazil; pacmf@usp.br

**Keywords:** melatonin, macrophages, beta-adrenergic receptors, immune-pineal axis, nuclear factor kappa B (NF-κB), extra-pineal melatonin synthesis

## Abstract

Melatonin (5-methoxy-*N*-acetylserotonin), the pineal hormone, is also synthesized by immune-competent cells. The pineal hormone signals darkness, while melatonin synthesized on demand by activated macrophages at any hour of the day acts locally, favoring regulatory/tolerant phenotypes. Activation of β-adrenoceptors in pinealocytes is the main route for triggering melatonin synthesis. However, despite the well-known role of β-adrenoceptors in the resolution macrophage phenotype (M2), and the relevance of macrophage synthesized melatonin in facilitating phagocytic activity, there is no information regarding whether activation of β-adrenoceptors would induce melatonin synthesis by monocytes. Here we show that catecholamines stimulate melatonin synthesis in bone marrow-derived dendritic cells and RAW 264.7 macrophages. Activation of β-adrenoceptors promotes the synthesis of melatonin by stimulating cyclic AMP/protein kinase A (PKA) pathway and by activating the nuclear translocation of NF-κB. Considering the great number of macrophages around sympathetic nerve terminals, and the relevance of this system for maintaining macrophages in stages compatible to low-grade inflammation, our data open the possibility that extra-pineal melatonin acts as an autocrine/paracrine signal in macrophages under resolution or tolerant phenotypes.

## 1. Introduction

Melatonin acts as a buffer of the immune system, stimulating immune responses under immunosuppressed conditions, and repressing innate and adaptive immune responses in physiological conditions [1]. In control conditions, nocturnal melatonin rise impairs leukocyte migration through the endothelial layer, controlling the entry of defense cells in healthy tissues [2]. During the mounting of an inflammatory response, pro-inflammatory cytokines and pathogen- or danger-associated molecular patterns (PAMPs and DAMPs) reduce or even suppress pineal gland melatonin synthesis [3,4], favoring the migration of leukocytes to the injured tissue. The duration of PAMPs/DAMPs inhibition of pineal melatonin synthesis depends on the nature of the stimuli [5,6,7,8]. The incubation of human colostrum monocytes with *Escherichia coli* induces melatonin synthesis which stops after all bacteria are killed, whereas in the same cell type the melatonin synthesis induced by the PAMP derived from the fungi membrane, zymosan, lasts throughout the experimental observation [7,8]. Both exogenous melatonin [9,10,11,12] and melatonin synthesized by activated immunocompetent cells regulate the inflammatory response [13,14,15,16,17,18]. In resolution macrophages (M2), melatonin induces the synthesis of membrane proteins, such as dectin-1 [6] which increases phagocytosis [5,6], and arginase I which deviates arginine conversion from nitric oxide into ornithine and urea [19]. These are transient changes, and the return to basal conditions results in the restoration of daily pineal melatonin rhythm and reduction of melatonin synthesis by macrophages. Such switching of melatonin sources, from pineal-to-immune-back-to-pineal, was defined as the immune-pineal axis, which provides a theoretical basis for the chronobiotic and protective functions of melatonin [3].

The daily rhythm of pineal melatonin synthesis relies on the stimulation of pinealocytes β-adrenoceptors which trigger the transcription of the arylalkylamine-*N*-acetyltransferase (*Aanat*) gene and the activation of the enzyme [20]. Both effects are mediated by cyclic AMP-induced protein kinase A (PKA) activation. The suppression of *Aanat* transcription induced by PAMPs and DAMPs is mediated by the nuclear translocation of the nuclear factor kappa B (NF-κB) [4], which is the key element for switching melatonin synthesis from pineal gland to immune-competent cells. Indeed, the dimer p50/p50 is responsible for suppressing *Aanat* transcription, while dimers containing RelA or c-Rel lead to the transcription of this gene [21]. Accordingly, p50/RelA activation during the pro-inflammatory phase enhances the transcription of Rel, the gene that encodes c-Rel, which in its turn increases the transcription of proteins involved in the regulatory phase [22]. As such, the dimers of NF-κB coordinate the site of melatonin synthesis at distinct phases of the immune-inflammatory response.

Given that pineal melatonin is a temporal marker, the on/off switch of biosynthetic enzymes is crucial. As mentioned above, upon activation, β-adrenoceptors located in pinealocytes trigger the cAMP/PKA/CREB pathway and lead to *Aanat* transcription. AANAT converts serotonin into *N*-acetylserotonin, which is then methylated by *N*-acetylserotonin methyltransferase (ASMT) originating melatonin (5-methoxy-*N*-acetylserotonin). Once translated, AANAT is rapidly ubiquitinated and degraded by the proteasome [20,23]. The stabilization of the enzyme by the chaperone 14-3-3 requires the phosphorylation of AANAT by PKA. In the pineal glands of nocturnal animals, both gene transcription and enzyme activation necessary for transforming serotonin into *N*-acetylserotonin are regulated by β-adrenoceptor activation while in humans (diurnal animals) the gene is constitutively transcribed [23]. Thus, in humans, the activation of the enzyme is the only step regulated by darkness.

Until now, only PAMPs and DAMPs, such as LPS and zymosan, were shown to induce the synthesis of melatonin in macrophages and lymphocytes [5,6,15,16,17,19]. Macrophages express β-adrenoceptors [24,25], which can be activated autocrinally by locally synthesized catecholamines, and/or by noradrenaline released by sympathetic nerve terminals [25,26]. Stimulation of α2 adrenoceptors potentiates tissue injury induced by PAMPs [24,25,26], while activation of β2-adrenoceptors reduces tumor necrosis factor [27,28], metalloproteinase-12 protein and monocyte chemoattractant protein-1 (MCP-1) synthesis [29]. Consequently, β2-adrenergic stimulation reduces macrophage [26] and neutrophil [27] mobility, favoring the predominance of macrophages with a resolution phase phenotype.

Despite the strong evidence that adrenergic signaling modulates macrophage function, and that adrenoceptor/cAMP/PKA is the pivotal pathway for triggering melatonin synthesis in pinealocytes, no study has addressed the possibility that activation of β-adrenoceptors induces the synthesis of melatonin by macrophages or other immunocompetent cells. In addition to macrophages [4,5,6], dendritic cells (DCs), which act as the link between the innate and adaptive immunity [30], are potential candidates for synthesizing melatonin. Here we show that β-adrenergic stimulation triggers the synthesis of melatonin in primary culture bone marrow-derived dendritic cells and the murine cell line RAW 264.7. By using different pharmacological tools, we disclosed the signaling pathway involved in melatonin synthesis in macrophages, evidencing the key role of the transcription factor NF-κB in underlying this phenomenon. The similarities and specificity of the signaling pathways involved in melatonin synthesis in pinealocytes and in macrophages are discussed, as well as their relevance for shuttling melatonin production from pineal to extra-pineal cells.

## 2. Results

**Figure 1:**
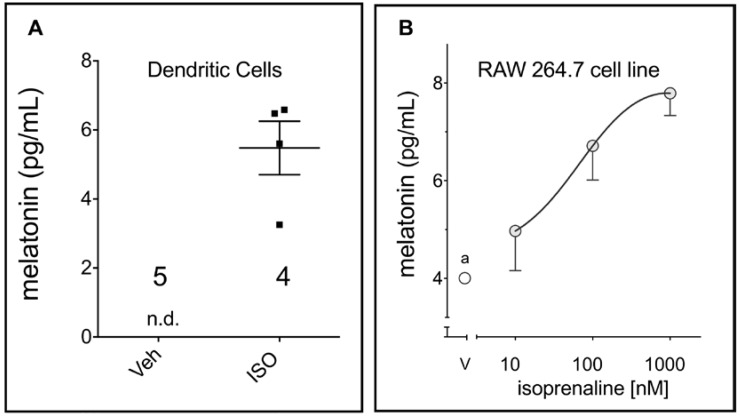
β-adrenoceptor triggers melatonin synthesis in phagocytes**.** (**A**) Bone marrow dendritic cells (isoprenaline, ISO, 10 µM, 24 h; n.d., not detected); the values for each replicate and the mean ± SEM are shown in the graph. (**B**) RAW 264.7 macrophages isoprenaline 3 h (a)—vehicle = 4.0 ± 0.01, the melatonin ELISA kit detection limit is quoted as 3–4 pg/mL). Data are shown as mean ± SEM of three independent experiments.

Figure 1 shows activation of β-adrenoceptors in DCs and RAW 264.7 by isoprenaline induced melatonin synthesis. No melatonin was detected in DC incubated with the vehicle. The amount of melatonin measured in the medium of RAW 264.7 line cells was just above the detection limit.

**Figure 2:**
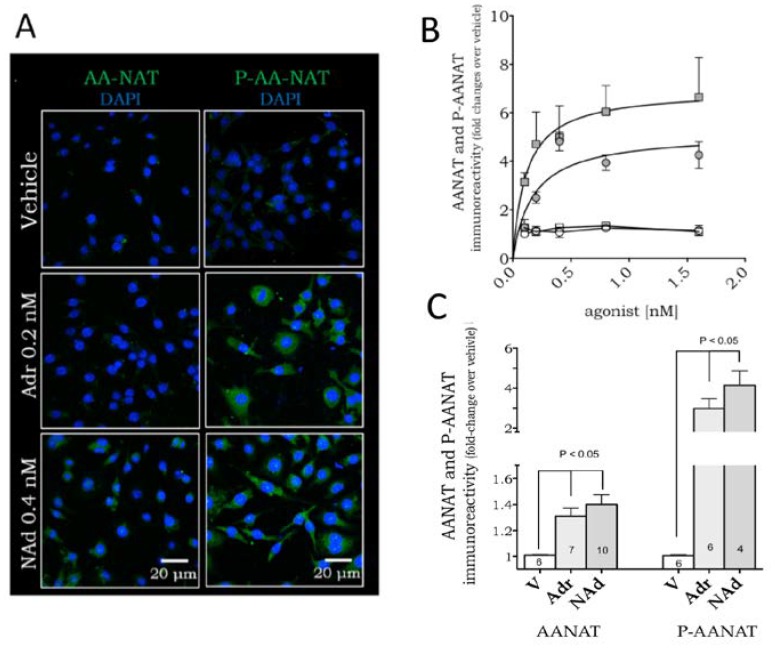
Adrenaline and noradrenaline induce expression of AANAT and P-AANAT in RAW 264.7 macrophages. (**A**) Representative immunofluorescence showing AANAT or P-AANAT expression in the cytoplasm of cells stimulated with adrenaline (Adr, 0.2 nM) or noradrenaline (NAd, 0.4 nM). The enzyme expression is shown in green, and the nuclei are stained by DAPI (blue); (**B**) Dose-response curve of AANAT (white symbols) and P-AANAT (grey symbols) expression in RAW 264.7 cells incubated with increasing concentrations of adrenaline (circles) or noradrenaline (squares); (**C**) The same data presented in (**B**), of AANAT and P-AANAT expression induced by adrenaline (0.2 nM) and noradrenaline (0.4 nM), compared to vehicle (V; 0.001% ethanol) and plotted as single concentrations to better compare the catecholamines effect on both enzymatic forms. Data are shown as mean ± SEM, normalized by the respective vehicle group. Data represent the mean of four to ten independent experiments.

We next evaluated the mechanism of action underlying β-adrenoceptor-induced melatonin synthesis by determining the expression levels of the key enzyme controlled by adrenergic stimulation in the pineal gland and the corresponding signaling pathways. Non-stimulated RAW 264.7 macrophages expressed low levels of AANAT and P-AANAT (Figure 2A). Similar to that observed in pinealocytes, both noradrenaline and adrenaline increased the protein levels of AANAT and P-AANAT in a dose-dependent manner (Figure 2B). As the increase in AANAT is much smaller (less than two-fold) than the increase in P-AANAT (four to five-fold), we also present the data in adjusted scales to show the statistical difference between the maximal responses of vehicle- and catecholamine-treated cells (Figure 2C).

**Figure 3:**
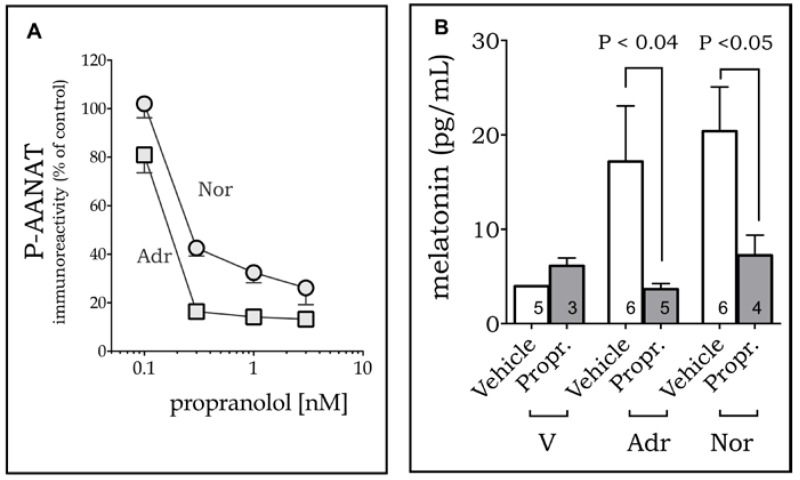
β-adrenoceptors mediate melatonin synthesis in RAW 264.7 macrophages. Propranolol (Prop.; 30 min) inhibits P-AANAT expression (**A**) and melatonin synthesis (**B**) induced by adrenaline (Adr; 0.2 nM; squares) and noradrenaline (Nor; 0.4 nM; circles). V = vehicle (0.001% ethanol). Data represent mean ± SEM (**A**)—each point is the mean of three to six independent experiments; (**B**)—the number of experiments is shown inside the bars).

The effect of adrenaline and noradrenaline on the induction of P-AANAT was mediated by β-adrenoceptors, as it was blocked by propranolol (Figure 3A). As P-AANAT is the stable and active form of the enzyme, we confirmed that melatonin synthesis follows P-AANAT levels by measuring the melatonin content released in the culture medium by cells treated or not with propranolol 30 min before the addition of adrenaline or noradrenaline (Figure 3B). The equieffective concentrations of adrenaline (0.4 nM) and noradrenaline (0.2 nM) were chosen for evaluating the effect of the antagonists, as they were the minimal concentration of each drug that attained the maximum response.

**Figure 4:**
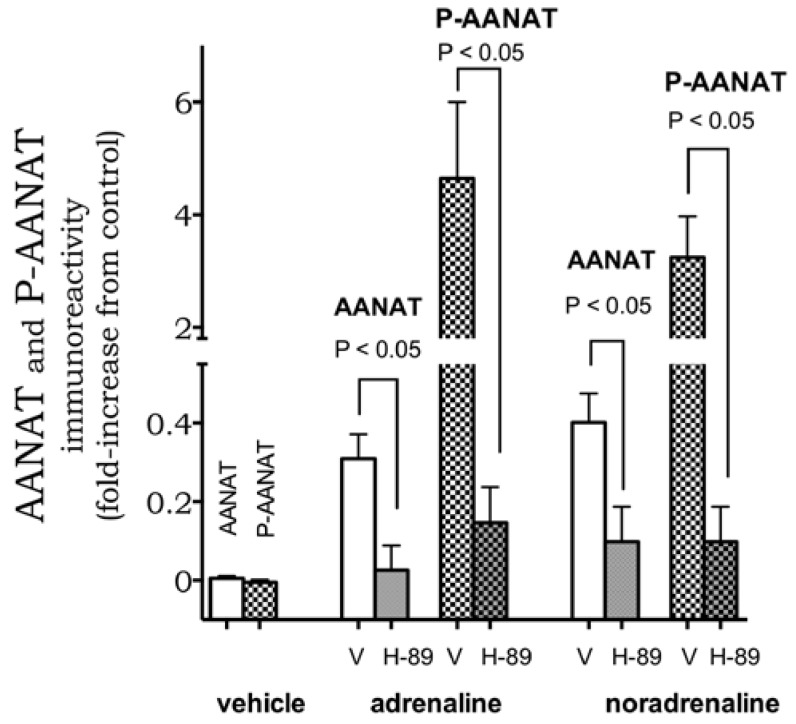
PKA activity modulates AANAT and P-AANAT expression. PKA inhibitor H-89 (H, 100 nM, 30 min) inhibits adrenaline (0.4 nM) and noradrenaline (0.2 nM)-induced increase in AANAT (open bars) and P-AANAT (crosshatched) immunoreactivity. Data represent mean ± SEM of three to six independent experiments. V = vehicle (0.001% ethanol).

To further characterize the system, we evaluated the signaling pathways involved in catecholamine-induced melatonin synthesis in macrophages. The two pathways tested were the cAMP/PKA pathway, known to trigger transcriptional and post-transcriptional regulation of *Aanat* in pineal glands, and the transcription factor NF-κB, which induces the transcription of Aanat in mouse macrophage cell line RAW 264.7 and in human colostral mononuclear cells [9,10,20]. Pre-incubation with the inhibitor of PKA, H-89 (100 nM, 30 min), blocked adrenaline- (0.2 nM) and noradrenaline (0.4 nM)-induced expression and phosphorylation of AANAT (Figure 4).

**Figure 5:**
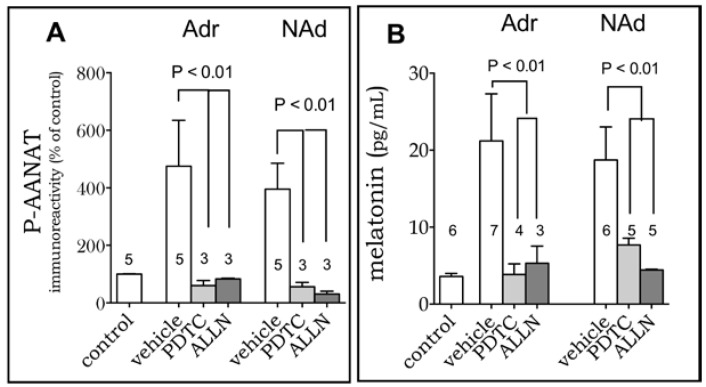
The transcription factor NF-κB mediates melatonin synthesis in RAW 264.7 cells. PDTC (P, 25 μM, 30 min) or ALLN (A, 50 μM, 30 min) blocked Adr and Nor-induced increase in the expression of PAANAT (**A**) and melatonin production (Adr, 0.2nM; NAd, 0.4 nM, 3 h). (**B**) Data are shown as mean ± SEM; the number of replicates is shown in the graph. P-AANAT expression was normalized by the vehicle group.

Inhibiting the binding of NF-κB dimers to DNA with PDTC (25 μM) completely abolished adrenaline- and noradrenaline-induced increase in P-AANAT and melatonin levels) (Figure 5). Similar results were obtained by inhibiting the degradation of IκBα (nuclear factor of kappa light polypeptide gene enhancer in B-cells inhibitor, alpha), which retains NF-κB dimers in the cytoplasm, by using ALLN (50 µM) (Figure 5). 

## 3. Discussion

The central question of this study was whether the activation of adrenoceptors could induce melatonin production by phagocytes, such as dendritic cells and macrophages, as observed in pinealocytes. Activated phagocytes are known to synthesize melatonin, which acts autocrinally enhancing the phagocytic ability and reducing the activity of enzymes involved in the pro-inflammatory phase of the defense response [6,31].

**Figure 6:**
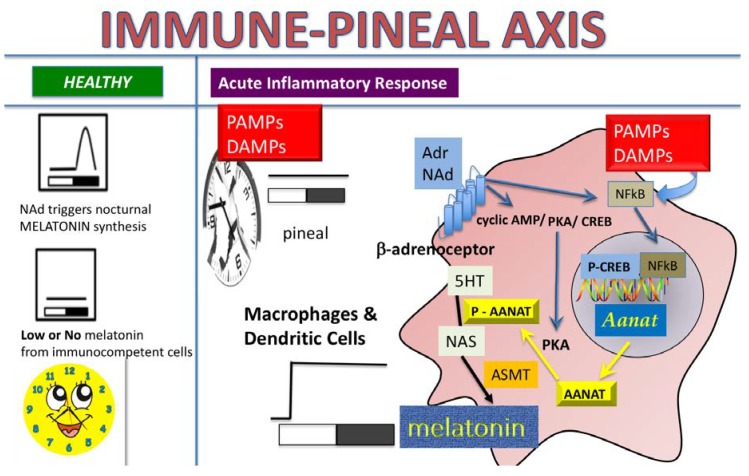
The Immune-Pineal Axis—Catecholamines induce melatonin synthesis in macrophages—Pathogen- and damage-associated molecular patterns (PAMPs and DAMPs), such as lipopolysaccharide (LPS), β-amyloid peptide and the pro-inflammatory cytokine tumor necrosis factor (TNF), block the synthesis of melatonin by pinealocytes. Reduction of nocturnal melatonin surge favors the migration of leukocytes to the site of the lesion. The same PAMPs/DAMPs induce the synthesis of melatonin in macrophages, microglia, and dendritic cells. This switch in melatonin source allows the migration of cells to the site of lesion, where, locally synthesized melatonin plays regulatory roles. Interestingly, activation of NFκB is the key mechanism for blocking/inducing melatonin synthesis in pinealocytes/macrophages, as it regulates the transcription of the gene coding the enzyme (AA-NAT) that catalyzes the conversion of serotonin in *N*-acetylserotonin, the immediate precursor of melatonin [4,34]. One of the open questions in this system was whether catecholamines might lead to melatonin synthesis in macrophages/monocytes as it does in the pineal gland. Here we showed that activation of β-adrenoceptors induces the transcription of *Aanat* in a PKA and NF-κB dependent manner.

Following the same mechanism of action observed in the pinealocytes, here we show that activation of β-adrenoceptors promotes the expression and phosphorylation of AANAT in phagocytes. The increase in P-AANAT levels was much more robust than that of AANAT, probably because most of the newly translated protein is immediately phosphorylated by the same β-adrenergic/PKA signaling pathway (Figure 6 summarizes the discussion). As observed in pinealocytes [23], the synthesis of melatonin induced by β-adrenoceptors activation depends on PKA, which phosphorylates AANAT in serine/threonine residues stabilizing the protein and allowing the conversion of serotonin into *N*-acetylserotonin [31,32]. Alternatively, this difference in AANAT/P-AANAT levels can be explained by the fact that the non-phosphorylated enzyme is ubiquitinated and degraded by the proteasome, while the phosphorylated enzyme binds to the chaperone 14-3-3, which hinders its degradation [33].

In contrast to AANAT, the transcription factor NF-κB has opposite effects in pinealocytes and macrophages. In rat pinealocytes, NF-κB nuclear translocation is rhythmic and synchronized to the environmental photoperiod information. The expression of the genes related to inflammatory responses presented a daily rhythm in the pineal gland of healthy rats [35,36]. At dusk, the expression of the genes encoding for toll-like receptors, cytokines, NF-κB subunits and related molecules, adhesion molecules, caspases and inducible nitric oxide synthase presented a very fast decrease between animals killed minutes before or after darkness [36]. The sympathetic induction of the transcription of *Aanat* and the synthesis of melatonin required the reduction of the nuclear content of NF-κB dimers, which occurs just after the lights turn off [35]. Interestingly, the gene that encodes for the NF-κB/RelA subunit, which regulates the expression of the genes that trigger the pro-inflammatory response, did not show a rhythmic variation during the day in healthy animals [36]. On the other hand, the increase in nuclear NFκB triggered by toll-like receptor 4 or tumor necrosis factor receptor impairs the transcription of *Aanat* [36,37,38,39] and the nocturnal peak of plasma melatonin [10]. In macrophages, both the adrenergic input and the nuclear translocation of NF-κB promote the synthesis of melatonin [3,22]. The explanation for this paradoxical response to NF-κB activation is that the dimers of NF-κB translocated to the nucleus of pinealocytes and macrophages are different. In the pinealocytes we observed the mobilization of p50/p50 dimers, which do not contain the transactivating domain, and therefore blocks *Aanat* transcription, while in macrophages the activated NF-κB dimers contain c-Rel, a transactivating domain positive subunit, which promotes the transcription of *Aanat* [22,36].

In macrophages, activation of β-adrenoceptors induces nuclear translocation of NF-κB, while the internalization of the complex β-adrenoceptors**/**β-arrestin upon receptor stimulation impairs the degradation of IκB by the proteasome and the nuclear translocation of NF-κB [39,40,41,42]. Thus, activation of β-adrenoceptors or sympathetic stimulation reduces the pro-inflammatory phenotype of macrophages. Indeed, β-arrestin2 inhibits excessive inflammation in infiltrated macrophages after myocardial infarction [43]. In U937 macrophages, noradrenaline induced increased IL-6 mRNA, while propranolol or PDTC reduced the production of IL-6 [44]. Besides regulating the expression of pro- and anti-inflammatory cytokines, here we show that the activation of β-adrenoceptors also leads to melatonin synthesis, which maintains the macrophages in an M2 phenotype.

Extra-pineal synthesis of melatonin by cerebellar cells decreases LPS-induced neuronal death [45], melatonin synthesis by human glioma cell lines is inversely correlated with tumor aggressiveness [46], and the increase in daytime plasma melatonin concentration is associated with faster surgical (laparotomy) wound-healing [47], reinforcing the anti-inflammatory role of locally synthesized melatonin. Recently, it was shown that neuronal cells also produce melatonin and, intriguingly, this production is located in the mitochondria [48]. Indeed, all the molecular machinery necessary for melatonin synthesis, including AANAT, ASMT and 14-3-3, was found in the mitochondria matrix, and brain-derived isolated mitochondria were able to convert serotonin into melatonin [48]. Whether melatonin synthesis and signaling by other cell types, such as macrophages and tumoral cells, also rely on mitochondrial location remains to be investigated.

Here we showed that stimulation of β-adrenoceptors induces melatonin synthesis in both BM-DCs and RAW 264.7 cells. Regarding the dendritic cells, it is well-known that these specialized phagocytes are responsible for bridging innate and adaptive immune responses [49], and that β-stimulation turns DCs to an anti-inflammatory state [50] and triggers T cell differentiation [51]. Indeed, sympathetic stimulation is essential for triggering the recovery phase of an immune response [52,53] in lymph nodes [54], bone marrow cells [55], and gut macrophages [56]. Melatonin produced on demand by immune cells [1,31] could represent a new player in the defense response not only directed to PAMPs and DAMPs but also mediated by activation of the sympathetic nervous system [57]. Taken together, our data provide further support to the new concept of endogenously and extra-pineal produced melatonin as an integral player in the function of phagocytes in situations of infection and/or sympathetic activation.

## 4. Materials and Methods

### 4.1. Drugs and Reagents

The reagents adrenaline, noradrenaline, isoprenaline, propranolol, H-89 (PKA inhibitor), the primary antibodies (anti AANAT: S0564; and anti-phospho AANAT: 0939), the secondary FITC-labelled antibody (S0814), and 4′,6-diamidino-2-phenylindole (DAPI) were purchased from Sigma-Aldrich (St. Louis, MO, USA). RPMI 1640 medium and fetal bovine serum were purchased from Gibco-Life Technologies (Grand Island, New York, NY, USA). The NF-κB blockers acetyl-l-leucyl-l-leucyl-l-norleucinal (ALLN) and pyrrolidine dithiocarbamate (PDTC) were purchased from Tocris (Minneapolis, MN, USA). The recombinant murine granulocyte–macrophage colony-stimulating factor (rmGM-CSF) were obtained from Peprotech (Rocky Hill, NJ, USA). All reagents were of analytical grade.

### 4.2. Mice

Eight- to ten-week-old BALB/c male mice were obtained from the Department of Immunology Animal Facility at the University of São Paulo and kept in micro-isolator cages under specific pathogen-free conditions. Experiments were performed following the guidelines for animal use and care approved by the Institutional Animal Care and Use Committee at the Institute of Biomedical Sciences of the University of São Paulo (CEUA–ICB/USP: n. 044, p. 31, b. 03/approved 27 May 2015).

### 4.3. Bone Marrow-Derived DC Generation and Culture

Bone-marrow-DCs were derived in vitro from bone marrow cells as previously described [58], and were characterized as CD11c/MHCII^+^ cells. Briefly, bone marrow cells were collected from the femur of BALB/c mice and cultured in RPMI 1640 medium (GIBCO, Grand Island, New York, NY, USA) supplemented with 10% heat-inactivated fetal bovine serum (FBS-GIBCO, Goiância, Brazil) and with antibiotic-antimycotic solution (GIBCO, Grand Island, New York, NY, USA). Cells were cultured for six days in tissue culture dishes (Becton Dickinson, Franklin Lakes, NJ, USA) in a total volume of 12 mL of medium with 20 ng/mL of recombinant murine granulocyte–macrophage colony-stimulating factor (rmGM-CSF). Half of the medium was replaced, and rmGM-CSF stimulation renewed on day 3 of culture. On day 6, non-adherent cells were collected and transferred to 24-well plates (2 × 10^6^ cell/well). Cells were treated with isoprenaline (10 nM) for 24 h and supernatants collected for melatonin measurement.

### 4.4. Cell Line RAW 264.7 Cultures

The in vitro studies were performed using the model of murine macrophage cell line RAW 264.7 cultured (37 °C, 5%, CO_2_) in RPMI 1640 medium supplemented with 10% FBS. The experiments were performed in cells plated at 2 × 10^6^ cells/well for melatonin measurement, and 2 × 10^5^ cells/well for immunofluorescence assays in medium without FBS.

### 4.5. Immunofluorescence Detection of the Enzyme AANAT

Adrenaline (Adr) and noradrenaline (NAd)-induced expression of AA-NAT and P-AA-NAT was evaluated in RAW 264.7 cells (2 × 10^5^ cells/well) plated in 8-well chamber slides (Nunc, Sigma-Aldrich, Rochester, NY, USA). The agonists at concentrations ranging from 0.1 nM to 1.6 nM were incubated for 15 min. The effect of the β-adrenoceptor antagonist, propranolol (0.1, 0.3, 1 and 3 nM), the PKA inhibitor (H-89; 100 nM), or the NF-κB inhibitors, [ALLN (50 µM) and PDTC (25 µM)] was tested against equieffective concentrations of adrenaline (0.2 nM) and noradrenaline (0.4 nM). The inhibitors were incubated for 30 min before adding the agonists, and the control cells were incubated with 0.001% ethanol in RPMI medium. After the incubation time, cells were fixed with methanol/acetone (1:1; 15 min at −20 °C), permeabilized with 0.5% saponin (10 min, room temperature) and incubated overnight (4 °C) with antibodies anti-AANAT (dilution 1:200) or anti-phospho-AANAT (dilution 1:300). The next day, the primary antibodies were revealed by the secondary FITC-labeled antibody (dilution 1:200, 1 h at room temperature). During the last five min, cell nuclei were stained with DAPI (1:1000). The slides were analyzed by confocal microscopy (Leica SP8 microscope, and an LSM 510, Carl Zeiss, Jena, Germany) using a 63 oil-immersion objective. FITC was excited using the 488 laser and the emitted fluorescence was measured at 525 nm. Images were taken from four randomly chosen fields in each well (≈30 cells/field), and the fluorescence intensity was quantified using ImageJ 1.41 software (http://rsb.info.nih.gov/ij).

### 4.6. Melatonin Dosage

Melatonin content was measured in the supernatant of RAW 264.7 macrophages or BM-DCs (both 2 × 10^6^ cells/well/500 μL) treated with adrenaline, noradrenaline or isoprenaline for 3 h. The blockers propranolol (0.3 nM), PDTC (25 M), or ALLN (50 M) were incubated for 30 min before adding the agonists (3 h). The experiments for detecting the expression of the enzymes required only 15 min incubation with the agonists, while for melatonin measurement a 3 h treatment was required. Melatonin was detected by commercial ELISA kit (Melatonin ELISA, IBL, Hamburg, Germany). The detection limit of the kit is 3.0–4.0 pg/mL.

### 4.7. Statistical Analyses

Data are expressed as mean ± SEM Groups were compared by one-way analysis of variance (ANOVA) followed by the Newman-Keuls post-test. *p* values lower than 0.05 were considered statistically significant. GraphPad Prism^®^ version 5.00 (GraphPad Software©, San Diego, CA, USA) was used for analysis of the data.

## 5. Conclusions

Melatonin is not only synthesized by the pineal gland and/or linked to environmental darkness. Activated immune-competent cells synthesize melatonin, which plays a role in regulating inflammatory responses. Here we showed that besides PAMPs and DAMPs, catecholamines also trigger melatonin synthesis in dendritic cells and macrophages. This effect is mediated by β-adrenoceptor/cAMP/PKA, which increases the expression of AA-NAT and its phosphorylation. Interestingly, as observed for PAMPs and DAMPs, catecholamines also trigger the NF-κB signaling pathway, which is responsible for *Aanat* transcription. Taken together, our data provide further support to the new concept of endogenously and extra-pineal produced melatonin as an integral player in the function of phagocytes in situations of infection and/or sympathetic activation.
